# Focus characterization of an X-ray free-electron laser by intensity correlation measurement of X-ray fluorescence

**DOI:** 10.1107/S1600577520009868

**Published:** 2020-08-17

**Authors:** Nami Nakamura, Satoshi Matsuyama, Takato Inoue, Ichiro Inoue, Jumpei Yamada, Taito Osaka, Makina Yabashi, Tetsuya Ishikawa, Kazuto Yamauchi

**Affiliations:** a Department of Precision Science and Technology, Graduate School of Engineering, Osaka University, 2-1 Yamada-oka, Suita, Osaka 565-0871, Japan; b RIKEN SPring-8 Center, 1-1-1 Kouto, Sayo, Hygo 679-5148, Japan

**Keywords:** Intensity correlation, X-ray focusing, focus characterization, X-ray fluorescence, X-ray free-electron lasers, SACLA

## Abstract

This paper proposes and demonstrates a simple method using the intensity correlation of X-ray fluorescence to evaluate the focused beam size of an X-ray free-electron laser (XFEL). This method was applied to the sub-micrometre focused XFEL beam at the SPring-8 Angstrom Compact Free Electron Laser, and the beam size evaluated using the proposed method was consistent with that measured using the knife-edge scan method. The proposed method is readily applicable to extremely small X-ray spots and can be applied for the precise diagnostics of sub-10 nm focused X-ray beams which have recently emerged.

## Abstract 2

A focus characterization method using the intensity correlation of X-ray fluorescence is proposed, and demonstrated for the sub-micrometre focused X-ray free-electron laser beam at the SPring-8 Angstrom Compact Free Electron Laser.

## 1. Introduction   

The recent advent of X-ray free-electron lasers (XFELs) (Emma *et al.*, 2010 ▸, Ishikawa *et al.*, 2012 ▸) has had a significant impact in various fields of X-ray science. One of the distinct characteristics of XFELs is their extremely high intensity. In combination with other advantageous characteristics such as ultrashort pulse duration (Inubushi *et al.*, 2012 ▸; Behrens *et al.*, 2014 ▸) and nearly full transverse coherence (Gutt *et al.*, 2012 ▸; Inoue *et al.*, 2015 ▸), XFELs enable the realization of several groundbreaking experiments, such as damage-free structure determination of protein molecules (Chapman *et al.*, 2011 ▸), generation of exotic states of matter via X-ray irradiation (Vinko *et al.*, 2012 ▸) and the capturing of ultrafast transient states in chemical reactions (Kim *et al.*, 2015 ▸). To enhance this unique capability of XFELs further, focusing optics have been intensively developed and applied to various types of experiments.

With regard to the SPring-8 Angstrom Compact Free Electron Laser (SACLA) (Ishikawa *et al.*, 2012 ▸), 1 µm focused XFEL beams generated using Kirkpatrick–Baez (KB) mirrors (Yumoto *et al.*, 2012 ▸) have been widely used for damage-free structure determination of protein molecules (Suga *et al.*, 2015 ▸; Nango *et al.*, 2016 ▸). Furthermore, explorations of nonlinear X-ray optical phenomena (Tamasaku *et al.*, 2014 ▸; Yoneda *et al.*, 2014 ▸; Ghimire *et al.*, 2016 ▸; Inoue *et al.*, 2016 ▸) have been performed using sub-micrometre focused XFEL beams generated using KB mirrors (Mimura *et al.*, 2014 ▸; Yumoto *et al.*, 2020 ▸). The most advanced focusing optic system at SACLA is a sub-10 nm focusing system based on a multilayer KB mirror, which was developed by combining sophisticated mirror fabrication and wavefront sensing techniques (Matsuyama *et al.*, 2018 ▸).

Although the focused beam size is the primary consideration when designing an experiment and analyzing the results, focus characterization of XFEL beams is not straightforward, especially for tightly focused (sub-10 nm) beams. One of the main difficulties is that the knife-edge scan method (Handa *et al.*, 2010 ▸), which is a common method for focus characterization, is not suitable for evaluating tightly focused XFEL beams because the intense X-ray beam evaporates the knife-edge. Furthermore, the shot-by-shot positional and pointing fluctuations of the focused XFEL beam, which result from the stochastic nature of the XFEL amplification processes, introduce difficulties in precise focus characterization. As an alternative, wavefront sensing has been widely used for focus characterization (Seaberg *et al.*, 2019 ▸). However, the information obtained from the wavefront measurement is not always appropriate for focus characterization because the wavefront measurement tends to include systematic errors (Inoue, Matsuyama *et al.*, 2018 ▸), which often lead to an inaccurate assessment of both optic misalignments and beam size estimates. Another possible candidate for focus characterization, ptychography, has attracted immense attention and is often used for characterizing wavefields in synchrotron radiation facilities (Kewish *et al.*, 2010 ▸). Despite the recent successful use of ptychography for fully characterizing 125 nm XFEL beams (Schropp *et al.*, 2013 ▸), a series of technical constraints such as ensuring the stable position of the beam and sample, and attenuation to prevent radiation damage, must be imposed for evaluation of tightly focused XFEL beams. Given these limitations, there is a pressing need to develop a diagnostic method that can be applied to tightly focused XFEL beams.

In this study, a simple and direct method was developed to evaluate the beam size of tightly focused XFEL beams using the X-ray fluorescence generated by irradiating XFEL pulses onto metal foils. This method was applied to the sub-micrometre focused XFEL beam at SACLA, and the results were compared with focus characterization by the knife-edge scan method.

## 2. Principles   

In the proposed method, a metal foil is placed at the focused beam position, and the spatial distributions of the X-ray fluorescence beneath the transmitted XFEL beam are measured shot-by-shot using a two-dimensional detector. Subsequently, the degree of intensity correlation at two separate positions (*r*_1_, *r*_2_) on the detector plane is evaluated using the two-point intensity correlation of the X-ray fluorescence, which is expressed as
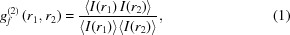
where *I*(*r*) is the intensity of the fluorescence at *r*, and the angle brackets represent the averages over different pulses. As the X-ray fluorescence is chaotic light, 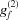 is related to the equal-time complex degree of coherence (Wolf, 2007 ▸; Inoue *et al.*, 2019 ▸) as
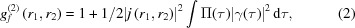
where 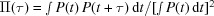 is the normalized autocorrelation function of the pulse envelope function of the fluorescence *P*(*t*) at the detector plane, and 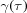 = 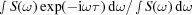 is the normalized Fourier transform of the power spectral density of the fluorescence *S*(ω). In the case when *r*_1_ = *r*_2_ ≡ *r*, the right-hand side of equation (2) contains an additional term 1/〈*I*_ph_(*r*)〉, where 〈*I*_ph_(*r*)〉 is the average number of detected photons per pulse at *r*, attributed to the photon statistics (Singer *et al.*, 2014 ▸; Inoue, Hara *et al.*, 2018 ▸; Trost *et al.*, 2020 ▸).

Let us consider a case in which the intensity distribution of an X-ray spot on the foil is described by a two-dimensional Gaussian function with standard deviations of 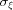 (horizontal) and 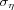 (vertical). According to the van Cittert–Zernike theorem, when *r*_1_ and *r*_2_ are located on the same plane perpendicular to the XFEL direction and are close to the beam axis, *j*(*r*_1_, *r*_2_) can be obtained as follows:
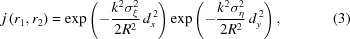
where *k* is the wavenumber, *R* is the distance between the foil and the detector, and *d*_*x*_ and *d*_*y*_ are the distances between *r*_1_ and *r*_2_ in the horizontal and vertical directions, respectively. When the detector is far from the beam axis, equation (3) is modified using the projection-slice theorem (Garces *et al.*, 2011 ▸) as
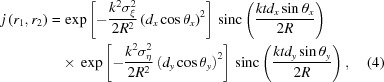
where *t* is the thickness of the foil, θ_*x*_ = arcsin(*L*_*x*_/*R*) and θ_*y*_ = arcsin(*L*_*y*_/*R*) are the angles of the detector, and *L*_*x*_ and *L*_*y*_ are the horizontal and vertical distances between the detector and the beam axis, respectively.

It should be emphasized that the proposed method is insensitive to fluctuations in the focused beam position and thus can be readily applied to small XFEL spots, such as a sub-10 nm focused beam, which gives a clear advantage compared with the use of the conventional knife-edge scan method.

In addition, it is useful to draw a comparison between the proposed method and a similar technique that uses speckle analysis of coherent scattering (Sikorski *et al.*, 2015 ▸, Inoue *et al.*, 2020 ▸). Coherent scattering allows for single-shot measurement and can handle long-pulse X-rays at synchrotron radiation facilities, whilst the proposed method cannot operate on a shot-by-shot basis and is available only for short pulse X-rays, the pulse lengths of which should be approximately comparable with the temporal coherent lengths of X-ray fluorescence (Inoue *et al.*, 2019 ▸). However, the proposed method has significant and unique advantages over coherent scattering, such as fewer assumptions involved in the analysis, and the simplification of both sample preparation and discrimination between fluorescence and excitation.

## 3. Experimental   

Based on the principles mentioned in Section 2, the beam size of the focused beam of 12 keV XFEL pulses at SACLA on beamline BL3 (Tono *et al.*, 2013 ▸) was evaluated. We used XFEL pulses with pulse energies of 585 ± 65 µJ at the optics hutch for the following analysis.

**Figure 1:**
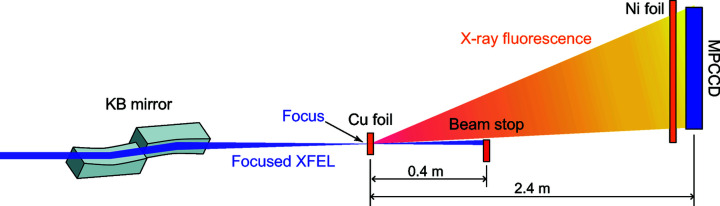
Schematic of the experimental setup.

Fig. 1 ▸ shows a schematic of the experimental setup. The XFEL pulses were focused using a total-reflection KB mirror system at Experimental Hutch 5 with the following parameters (Yumoto *et al.*, 2020 ▸): numerical aperture of 1.0 × 10^−3^ (vertical) and 2.0 × 10^−3^ (horizontal), grazing incidence angle of 4.0 mrad (vertical) and 3.8 mrad (horizontal), focal length of 0.50 m (vertical) and 0.24 m (horizontal), mirror length of 250 mm, estimated demagnification of 440 (vertical) and 918 (horizontal), and distance of 220 m from the source.

To measure the beam size beneath the focus, copper (Cu) foils with a thickness of 10 or 20 µm were used to generate X-ray fluorescence while changing the position of the foil along the X-ray beam axis. A dual-type multi-port charge-coupled device (MPCCD) detector (Kameshima *et al.*, 2014 ▸), located 2.4 m downstream of the focus at angles of θ_*y*_ = 23.5 mrad and θ_*x*_ = 4.6 mrad against the beam axis, was used to determine the shot-by-shot spatial distribution of the Cu *K*α fluorescence. Here, the Cu foil was scanned to allow the XFEL pulses to irradiate a fresh surface. A beam stop was installed to prevent irradiation of the transmitted 12 keV XFEL pulses on the detector. Furthermore, a 20 µm-thick nickel (Ni) foil was placed upstream of the detector to suppress the intensity of the *K*β fluorescence impinging on the detector. The elastic scattering lights were also suppressed by the Ni and Cu foils, resulting in near-invisible light levels. In addition, the XFEL intensity was reduced using silicon attenuators to prevent coherent emission of the fluorescence (Yoneda *et al.*, 2014 ▸) to [;ensure that equation (4) could be applied to the measured intensity correlation of the X-ray fluorescence. For each position of the Cu foil along the beam axis, ∼10^3^ fluorescent images were used for the analysis. Moreover, to validate the proposed method, the beam sizes were evaluated using the knife-edge scan method with a gold wire (diameter of 200 µm) to provide reference data.

## 4. Results and discussion   

**Figure 2:**
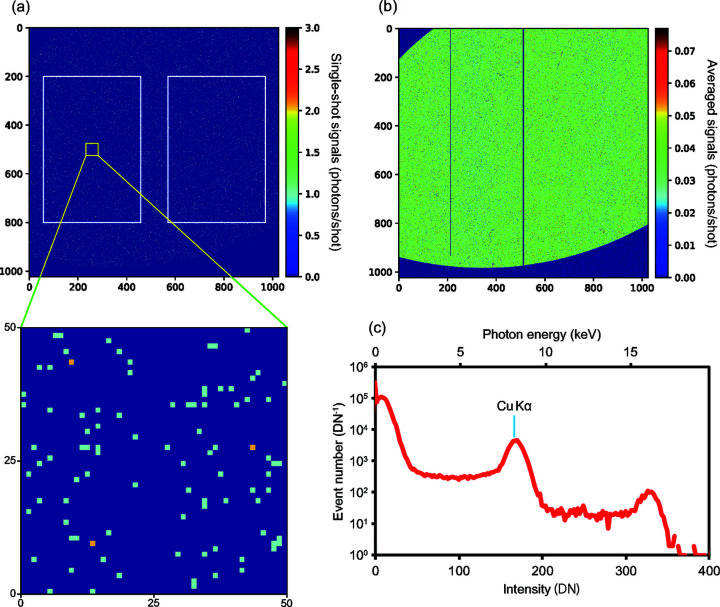
(*a*) Single-shot and (*b*) averaged spatial distributions of the number of detected Cu *K*α fluorescent photons. (*c*) A signal intensity histogram of multiple single-shot images. ‘DN’ in the axis title represents the digital number that is a raw value output from the MPCCD. The two white rectangles shown in (*a*) represent the analysed regions. In (*a*), the target area was divided as the centre region has a joint line of the two CCD elements.

Fig. 2 ▸ shows the single-shot and averaged spatial distributions of the Cu *K*α fluorescent photons detected using the MPCCD and the signal intensity histogram of multiple single-shot images. Using the shot-by-shot fluorescence signal within the rectangle areas shown in Fig. 2 ▸(*a*), the intensity correlation function of the Cu *K*α fluorescence was evaluated using the following equation including the Fourier transform,
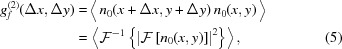
where the angle brackets represent the averages over pulses and pixels, and *n*_0_(*x*, *y*) is the number of detected *K*α fluorescent photons at the pixel (*x*, *y*) normalized to the average value over all the pulses. 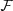 and 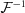 represent the Fourier transform and its inverse transform, respectively.

**Figure 3:**
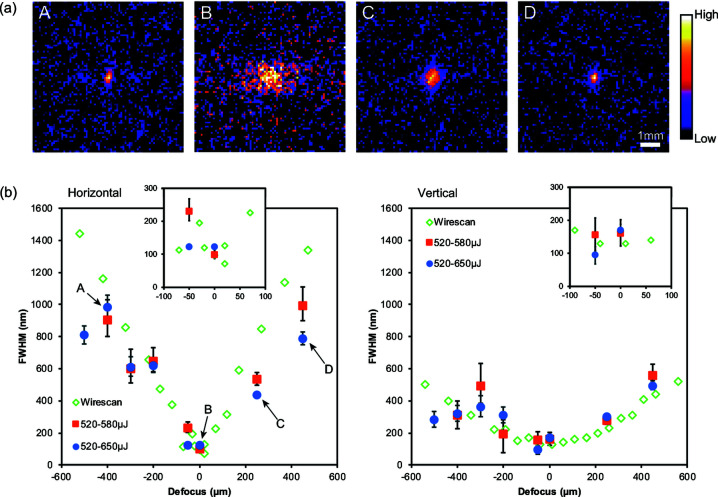
(*a*) Intensity correlation functions. (*b*) Beam size in FWHM at different positions beneath the focus. The inserted small graphs represent the beam size close to the focus. The intensity correlation functions in (*a*) are labelled A to D. The vertical and horizontal beam sizes at the corresponding defocus positions were estimated from the same intensity correlation function.

At each foil position along the beam axis, the intensity correlation function for small values of Δ*x* and Δ*y* is larger than unity [Fig. 3 ▸(*a*)], indicating that the intensity of the fluorescence is clearly correlated. By fitting the Gaussian function defined in equation (3) to the intensity correlation function, the full widths at half-maximum (FWHMs) of the function were obtained. Subsequently, the function FWHMs were converted to FWHMs of the beam using a calibration curve calculated using equation (4) [Fig. 3 ▸(*b*)]. To analyze the beam size carefully, different thresholds (520–580 µJ and 520–650 µJ) were employed to screen the XFEL pulses and the respective FWHMs of the beams were estimated, as previous work (Sikorski *et al.*, 2015 ▸) reported the possibility of shot-by-shot beam size fluctuation in relation to pulse energy fluctuation.

The evaluated beam sizes are consistent with the results of focus characterization realized using the knife-edge scanning method, which supports the validity of focus characterization realized using the proposed intensity correlation technique. In addition, it was concluded that the beam size variation with respect to the pulse energy variation is within the range of the measurement error and therefore had a negligible influence in this experiment. Moreover, the number of detected fluorescence photons was low, which resulted in a low signal-to-noise ratio and a relatively large error bar. To allow a more accurate determination of the beam size, the experimental parameters, including the shot number, Cu foil thickness and area of the detector, should be optimized. Along with the issue of the error bars, some deviations from the results of the knife edge scan become evident. It is assumed that this problem is caused by the measurement error of the proposed method.

## 5. Summary   

A simple method of focus characterization of XFEL beams using fluorescence has been developed. The proposed method has been applied to the sub-micrometre focused XFEL beam at SACLA. The evaluated beam size was consistent with that obtained using the conventional knife-edge scan method, thus validating the proposed method.

The proposed method can realize precise focus characterization of very tightly focused XFEL beams, because it can overcome critical problems such as fluctuations in the incident XFEL position, vibration of optical elements and radiation damage, which become more serious with a decrease in beam size.
